# Neuroinflammation is dependent on sex and ovarian hormone presence following acute woodsmoke exposure

**DOI:** 10.1038/s41598-024-63562-2

**Published:** 2024-06-06

**Authors:** Kartika Wardhani, Sydnee Yazzie, Onamma Edeh, Martha Grimes, Connor Dixson, Quiteria Jacquez, Katherine E. Zychowski

**Affiliations:** 1 College of Nursing, University of New Mexico-Health Sciences Center, Albuquerque, NM 87131 USA; 2 Biochemistry and Biotechnology (B-TEK) Group, Bioscience Division, Los Alamos National Laboratory, Los Alamos, NM 87545 USA; 3 Department of Pharmaceutical Sciences, College of Pharmacy, University of New Mexico-Health Sciences Center, Albuquerque, NM 87131 USA

**Keywords:** Wildfire, Sex-differences, Menopause, Toxicology, Inflammation, Inhalation, Immunology, Neuroscience, Environmental sciences

## Abstract

Woodsmoke (WS) exposure is associated with significant health-related sequelae. Different populations can potentially exhibit varying susceptibility, based on endocrine phenotypes, to WS and investigating neurological impacts following inhaled WS is a growing area of research. In this study, a whole-body inhalation chamber was used to expose both male and female C57BL/6 mice (*n* = 8 per group) to either control filtered air (FA) or acute WS (0.861 ± 0.210 mg/m^3^) for 4 h/d for 2 days. Neuroinflammatory and lipid-based biological markers were then assessed. In a second set of studies, female mice were divided into two groups: one group was ovariectomized (OVX) to simulate an ovarian hormone-deficient state (surgical menopause), and the other underwent Sham surgery as controls, to mechanistically assess the impact of ovarian hormone presence on neuroinflammation following FA and acute WS exposure to simulate an acute wildfire episode. There was a statistically significant impact of sex (*P* ≤ 0.05) and statistically significant interactions between sex and treatment in IL-1β, CXCL-1, TGF-β, and IL-6 brain relative gene expression. Hippocampal and cortex genes also exhibited significant changes in acute WS-exposed Sham and OVX mice, particularly in TGF-β (hippocampus) and CCL-2 and CXCL-1 (cortex). Cortex GFAP optical density (OD) showed a notable elevation in male mice exposed to acute WS, compared to the control FA. Sham and OVX females demonstrated differential GFAP expression, depending on brain region. Overall, targeted lipidomics in phosphatidylcholine (PC) and phosphatidylethanolamine (PE) serum and brain lipids demonstrated more significant changes between control FA and acute WS exposure in female mice, compared to males. In summary, male and female mice show distinct neuroinflammatory markers in response to acute WS exposure. Furthermore, ovarian hormone deficiency may impact the neuroinflammatory response following an acute WS event.

## Introduction

Woodsmoke (WS) exposure is undoubtedly a growing public health concern^1,2^. Climate change has contributed to increased incidence and severity of wildfires, while indoor WS exposure is a common public health issue, in sub-Saharan Africa and the developing world^3,4^. Identifying biological susceptibility to wildfire and WS exposures is important to eventually mitigate air pollution-induced disease and protect vulnerable populations. The full-scope of sex-dependent biological differences have yet to be determined following acute WS exposure. Wildfire events may impact the sexes differently. For example, wildfire smoke exposure has been noted to impact male infants born in the surrounding geographic area, more so than female infants. For example, there is an increase in macrosomic infants in impacted wildfire geographic area compared to normal-weights in male infants^5^. Estradiol presence has been implicated to trigger unique immunological responses in asthmatic, ovariectomized female mice^6^. Several air pollution studies have examined the role of sex-dependent air pollution impacts regarding lung, pulmonary and nasal biology. Nasal mucosa is one organ system implicated in driving sex-dependent impacts. In a randomized, controlled clinical study, with 2-h controlled chamber WS exposure, where exposure levels to PM were controlled (500 μg/cm^3^), IP-10 protein levels were suppressed by WS exposure^7^. Woodsmoke particle exposure prior to COVID infection results in altered gene expression in human nasal epithelial cells in a sex-dependent manner^8^. In previous studies, inhaled mixed vehicular emissions (MVE) decreased cerebral estrogen receptor-α mRNA expression^9^. In the same study, mixed vehicular emissions exacerbated demyelination in ovariectomized ApoE^−/−^ female mice^9^.

Sex differences have been noted in mouse models exposed to other air pollutants such as ozone^10,11^. Ozone exposure triggers differential immune mechanisms in asthmatic mice^10^ with male mice demonstrating an increased expression of immune response genes in the lung. Ozone also drives an increase in bioactive lipid mediators in females, compared to males^11^. Sex-specific differences have been noted in inflammation resolution following exposure to repetitive agricultural dust exposure^12^. Sex-specific differences to air pollution exposures have also been noted in child neurodevelopment, with respect to temporal associations on different cognitive domains^13^.

Although airway and lung biology has been explored with regard to air pollution biology and sex-differences, less is known about systemic immune and neuroimmune outcomes and mechanisms. Mixed vehicular emissions (MVE) has demonstrated demyelination in the brains of female ApoE^−/−^ mice^9^, which may influence central nervous system (CNS) diseases such as multiple sclerosis. This same study indicated that MVE decreased cerebral estrogen receptor- α, in female mice. Air pollution exposures during pregnancy have shown an increased autism spectrum disorder (ASD) in children, with a stronger association in boys^14^. Early postnatal exposure to ultrafine particulate matter results in neurochemical disruption and glial activation specifically in male mice^15^. Dendritic morphology varies according to sex and in vitro sex differences in dendritic morphology are driven by estrogen-dependent mechanisms^16^.

Lipid mediators may impact health and disease^17,18^. Phosphatidylethanolamine (PE) and phosphatidylcholine (PC) are the two most common glycerophospholipids and play a crucial role in cellular membrane development^19–21^. There are several immune-mediated diseases that have shown an alteration in lipid metabolism, more specifically the Kennedy pathway, that is responsible for de novo synthesis of PE and PC lipids^21^. Previously, our group has determined phenotypic alterations following particulate matter (PM) exposure in mice, specifically changes in PE, PC and triglycerides (TG) in isolated, serum-borne extracellular vesicles (EVs)^22^. In addition, PC is a phospholipid providing arachidonic acid for prostacyclin synthesis in thrombin-stimulated endothelial cells^23^.

**Figure 1:**
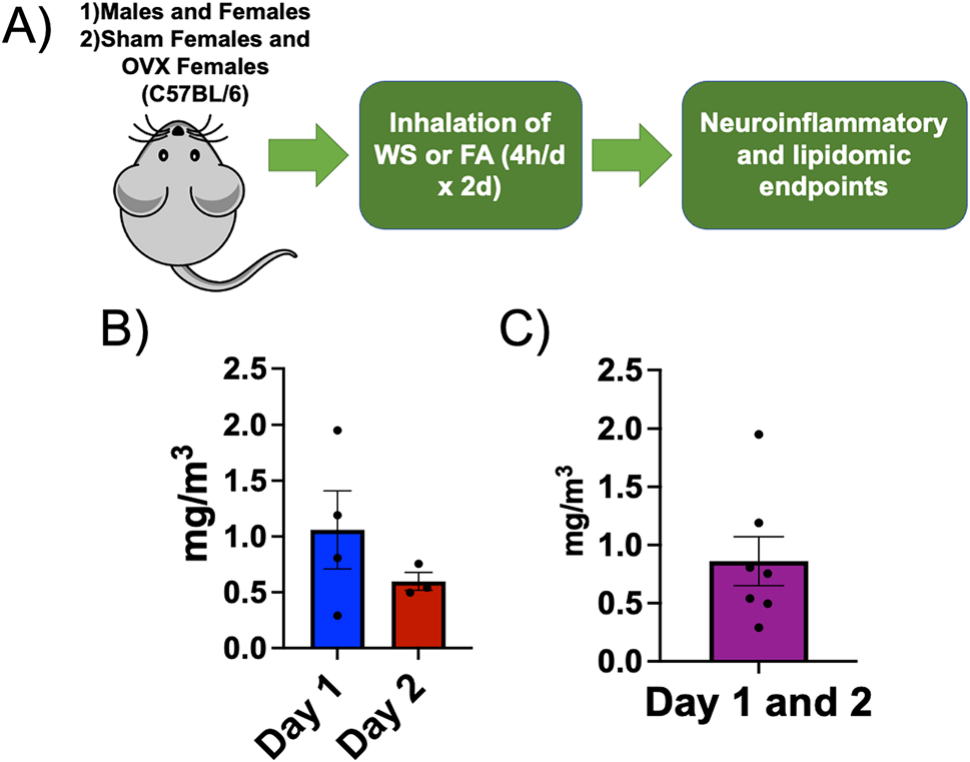
Graphical abstract. (**A**) Male and female C57BL/6 mice were exposed to either acute woodsmoke (WS) or control HEPA-filtered air (FA) for a 2-day exposure protocol with 4-h periods, daily (*n* = 8 per group). In a second set of experiments, Sham-operated and OVX female mice (6–8 weeks old) were then exposed to the same experimental conditions. (**B**) Typical average WS conditions (concentration ± standard error) for Day 1 (1.059 ± 0.349 mg/m^3^) and Day 2 (0.597 ± 0.080 mg/m^3^) during the acute WS exposures. (**C**) Combined aggregated concentrations for Day 1 and Day 2 overall (0.861 ± 0.210 mg/m^3^).

In this study, male and female C57BL/6 mice were used as in vivo models to assess sex-differences in neuroinflammatory and lipidomic impacts following an acute WS event. In a separate set of experiments within this same study, ovariectomy (OVX) was performed on female C57BL/6 mice to assess the mechanistic role of ovarian hormone presence following acute WS exposure, using a whole-body inhalation, WS system. Each experimental group was exposed to a 2-day protocol, which consisted of daily 4-h intervals under both HEPA-filtered air (FA, the control setting) and acute WS conditions (Fig. 1). This systematic approach enabled a thorough investigation, identifying potential systemic and neuroinflammatory biomarkers susceptible to influence by endocrinological phenotypes.

## Materials and methods

### Animals and ovariectomy (OVX) surgery

Male and female C57BL/6 mice, as well as female Sham and ovariectomy (OVX) mice (*n* = 8 per treatment group) were purchased at 6–8 weeks of age from Taconic Biosciences (Albany, NY, USA). This age range was chosen for its suitability in biomedical research due to the well-characterized nature of the strain, reaching sexual maturity, and minimizing experimental variability^24^. Using C57BL/6 mice aged 6–8 weeks offers a robust and consistent model for investigating sex hormone effects on immune responses^24^. In a second set of experiments, female C57BL/6 mice were subjected to OVX, or Sham surgery (*n* = 8 per treatment group). These procedures adhered to the approved protocol of the university's Institutional Animal Care and Use Committee (IACUC). In addition, this study was conducted in accordance with ARRIVE guidelines^25,26^. Mice were anesthetized with isoflurane, and buprenorphine (0.01 mg/mL) was subcutaneously administered to both the right and left sides of the abdomen (0.2 mL/mouse in total). The incision site was sterilized with ethanol, followed by a betadine/iodine swab, after which the skin was delicately separated, connecting it with the back tissues using a blunt dissection technique. The ovaries were removed, fat pads were replaced, and internal structures were placed in the original positions with a single stitch used to close the peritoneal incision. Mice were then returned to the recovery cage and vigilantly observed for a total of 10 days post-surgery for signs of distress and their incision recovery progress.

### Whole-body inhalation exposures

A whole-body, bench-scale exposure system was used for all rodent exposures^22^. TSI monitoring was used to determine particulate concentration in the exposure chamber and gravimetric filter samples (*n* = 3 per group) were collected to confirm true mass calculations. After a post-operative recovery phase and a 2-week acclimation period in suitable housing facilities, in accordance with approved IACUC procedures, all mice, including both male and female C57BL/6 mice, as well as female C57BL/6 mice subjected to Sham or OVX procedures, were moved to inhalation exposure chambers for acute WS or FA. They were subsequently assigned to a 2-day exposure regimen with daily 4-h intervals of acute WS at a concentration of approximately 0.861 ± 0.210 mg/m^3^ over 2d. Specifically, Day 1 had a chamber concentration of 1.059 ± 0.349 mg/m^3^ and Day 2 a concentration of 0.597 ± 0.080 mg/m^3^. Additionally, the control condition involved exposure to HEPA-filtered air (FA) in a HEPA-FA chamber, for 4 h/d for 2d (*n* = 8 per group, Fig. 1). Woodsmoke exposures consisted of a 2-g burned biomass wood sample, sourced from local trees in the Southwestern United States.

### Gene expression in brain hemispheres samples

Gene expression on isolated lung lobe and brain hemisphere samples was assessed using real-time quantitative polymerase chain reaction (RT-qPCR). Lung and brain tissue were dissected from recently euthanized mice, rapidly snap-frozen with liquid nitrogen, and stored in a 193.15 K freezer until they were prepared for RT-qPCR analysis. Total RNA was extracted using a commercial RNA kit (RNeasy, Qiagen, Germantown, MD, USA), followed by reverse transcription of 500 ng of the isolated RNA using a High-Capacity cDNA Reverse Transcription Kit (Applied Biosystems, Foster City, CA, USA) before qPCR acquisition. The resulting cDNA samples were diluted 1:4 with nuclease-free water. Ribosomal gene 18 s rRNA was used as a housekeeping gene for both lung and brain tissue. Gene expression was assessed using SYBR green expression protocols as per manufactures recommendations for iTaq Universal SYBR Green Supermix (BioRad, Hercules, CA, USA) and a 384 CFX Opus Real-Time PCR System (Bio-Rad, Hercules, CA, USA). Gene target analysis was conducted using Quanti-Tect mouse primers (Qiagen, Redwood City, CA, USA) included *IL-1b* (QT01048355), *CCL-2* (QT00167832, *TNF-a* (QT00116564), *CXCL-1* (QT00115647), *CCL-5* (QT01747165), *TGF-b* (QT00145250), *IL-6* (QT00098875) and 18s rRNA (QT02448075), for various brains and brain regions. Relative gene expression was then analyzed using the 2 − ∆∆CT method^27^, and FA exposure groups were normalized to 1.

### Glial fibrillary acidic protein (GFAP) staining

Brains were dissected from each mouse and the right brain hemisphere was isolated. The right hemisphere was embedded using an optimal cutting temperature (OCT) media. Following freezing using dry ice and ethanol, the block was sectioned (30–60 µm) using a standard cryostat (Leica, Wetzlar, Germany) and slices were mounted on positively charged slides. Glial fibrillary acidic protein (GFAP) rabbit polyclonal antibody (Abcam, Cambridge, MA, USA) was diluted to 1:2000 and pipetted onto each individual slide and incubated overnight at 277.15 K. The next day immunoglobulin G (IgG) was diluted to 1:1000 and pipetted onto sections to stain for 2 h. Sections were counterstained with DAPI (Thermo Scientific, Waltham, MA, USA) and subsequently stored in a sealed box protected from light for 8 min. Prolong Gold antifade reagent (Invitrogen, Waltham, MA, USA) was then applied to the tissue. Slides were then stored at 293–298 K (room temperature).

### Microscopic imaging of brain regions

Hippocampus and cortex images were taken using Leica TSC SP8 Confocal Microscope. DAPI and GFAP were captured at excitation peaks of 359 nm and 490 nm, and emission peaks at 457 nm and 535 nm, respectively, using a 20 × oil objective with a 2 × zoom (resulting in 80 × magnification) to image the slides. The cortex was imaged in accordance with previous studies on inhaled PM^22^, with 3 images captured per slide to visualize both nuclei and reactive astrocytes within this brain region. A representative image was then selected for each treatment group, as depicted in Figs. 5, 6 and Supplemental Fig. S1.

### HALO image analysis of reactive astrocytes

High-throughput, machine-learning based astrocyte quantification using HALO Analysis (Indica Labs, Albuquerque, NM, USA) was used to assess total astrocyte area (µm^2^), optical density (OD), percentage area, inner and outer area of the astrocyte, minimum and maximum diameter and percentage of GFAP stained cells colocalized with DAPI. After the annotated algorithm was established, the images were processed as a cohesive set, following the previously described method^22^. Astrocytic assignment and automated analysis are further visualized in Supplemental Fig. S1.

### Targeted lipidomics and liquid chromatography–mass spectrometry (LC–MS)

Brain and plasma samples were weighed or aliquoted then snap-frozen and shipped to the Wayne State University Lipidomics core for further analysis. Samples were homogenized using 1 mL phosphate buffered saline (50 mmol/L phosphate containing 0.9% sodium chloride) using a high-frequency oscillator. The homogenates were centrifuged at 6000* g* for 10 min, and the resulting supernatant was used for lipid extraction with C18 solid-phase extraction cartridges, following the previously described procedure^28–30^. Lipids, specifically phosphatidylcholine (PC) and phosphoethanolamines (PE), were analyzed using LC–MS, with a detection limit capable of detecting substances as low as 1 pg on the column and a quantitation limit of 5 pg on the column, while maintaining a signal-to-noise ratio > 3. Tissue weights consistently measured at 15 mg were used to normalize the data, reported as ng per gram (ng/g) of tissue. Lipids were subsequently validated using a commercially-available ELISA (Item No. 10009926, Cayman Chemical, Ann Arbor, MI, USA), as outlined in Supplemental Fig. S2. In addition, lipid panels were validated against internal commercially-available standards in the Wayne State Lipidomics Core facility.

### Statistical analysis

These studies were designed as 2 × 2 factorial two-way analysis of variances (ANOVAs) with sex (C57BL/6 males versus females) or surgery (Sham-operated versus OVX females) as the first factor, and treatment (the control FA group versus group exposed to acute WS) as the second factor. Statistical significance was determined to assess the impact of the following factors: sex, surgery, treatment, and interactions between sex and treatment or between surgery and treatment, on neuroinflammation, including brain mRNA gene expressions, and lipidomics in the plasma and brain. These interactions were examined using a 2 × 2 factorial ANOVA, followed by Tukey's post-hoc tests, which identified statistically significant mean differences between groups. The results were presented as mean normalized values ± standard error of the mean (SEM), with statistical significance set at *P*-values < 0.05. In experiments where only two variables were compared (for example the control FA group versus group exposed to acute WS), a Student’s T-test was executed to determine statistical significance (*P* > 0.05). Data were graphed using GraphPad Prism software (GraphPad Holdings, LLC, San Diego, CA, USA), when appropriate.

### Ethical approval and consent to participate

This study was approved by the University of New Mexico’s IACUC, Animal Welfare Assurance: D16-00228 (A3350-01), USDA Registration # 85-R-0014. This study was conducted in accordance with ARRIVE guidelines^25,26^.

## Results

### Sex and OVX surgery impacts on brain mRNA gene expression following acute WS exposure

**Figure 2:**
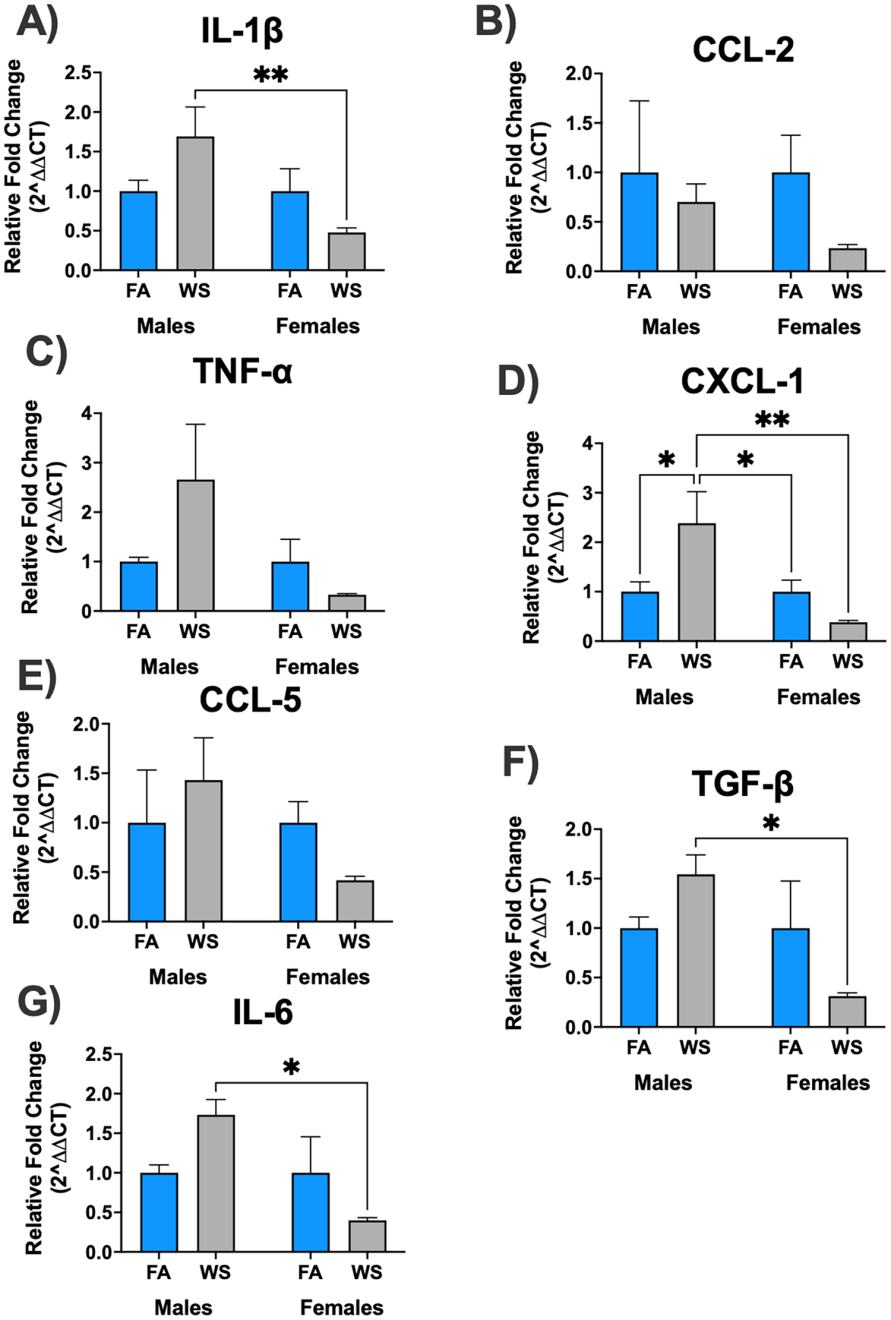
Sex-dependent brain mRNA gene expression levels in response to acute WS exposure (**A-G**). Brain mRNA relative gene expression levels demonstrated a response influenced by sex when exposed to acute WS. The data were analyzed using a two-way ANOVA, and the *P*-values for each variable (sex, treatment, and interactions between sex and treatment) on brain mRNA genes, where *P* < 0.05 signified statistical significance, were presented in the Supplemental Table S1(a). Asterisks denoted statistically significant mean differences between groups, as identified by Tukey’s post-hoc test (*P < 0.05; **P < 0.01). Data were presented as mean normalized values ± SEM. Each treatment group consisted of *n* = 8 mice.

**Figure 3:**
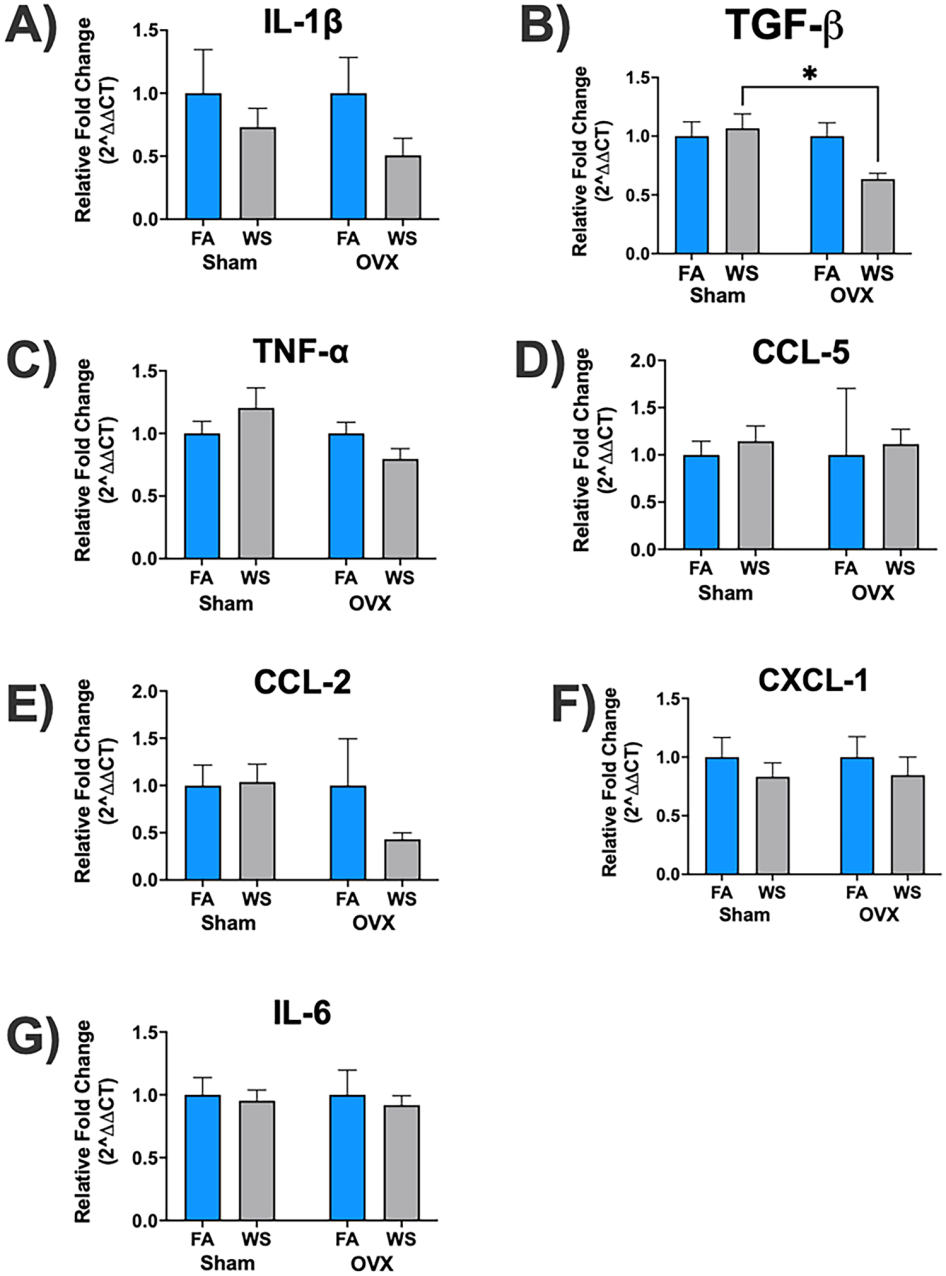
Ovariectomy (OVX)-dependent effects on mRNA gene expression levels in the hippocampus following acute WS exposure (**A-G**). Analysis was conducted using a two-way ANOVA from control FA and acute WS exposures in Sham-operated and OVX female mice. The *P*-values for each variable (surgery, treatment, and interactions between surgery and treatment) in relation to hippocampal mRNA gene expression levels, where *P* < 0.05 signified statistical significance, were presented in the Supplemental Table S1(b). Asterisks denoted statistically significant mean differences between groups, as identified by Tukey’s post-hoc test (*P < 0.05). Data were expressed as mean normalized values ± SEM. Each treatment group had *n* = 8 mice.

**Figure 4:**
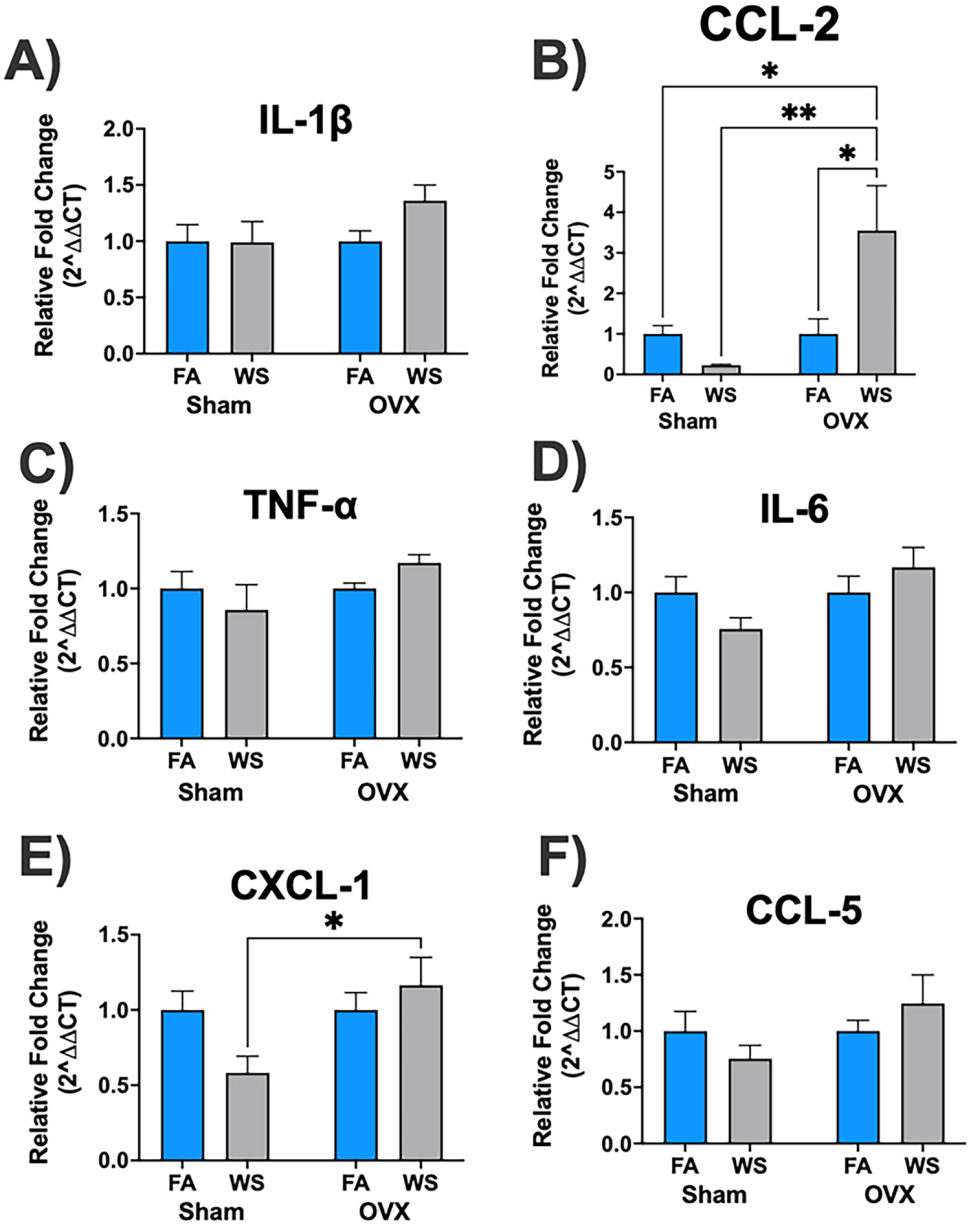
Ovarian hormone-dependent responses of cortical mRNA gene expression to acute WS exposure (**A-F**). Analysis was performed using a two-way ANOVA comparing control FA and acute WS exposures in Sham-operated and OVX female mice. The *P*-values for each variable (surgery, treatment, and interactions between surgery and treatment) concerning mRNA gene expression in the cortex, where *P* < 0.05 indicated statistical significance, were presented in the Supplemental Table S1(c). Asterisks denoted statistically significant mean differences between groups, as identified by Tukey’s post-hoc test (*P < 0.05; **P < 0.01). Data were reported as mean normalized values ± SEM. Each treatment group had *n* = 8 mice.

Woodsmoke (WS) exposure acute effects on sex-dependent and ovariectomy (OVX) surgery responses in brain mRNA relative gene expression were investigated (Figs. 2, 3, 4). Assessment of brain mRNA gene expression in male and female C57BL/6 mice revealed diverse patterns in response to acute WS exposure (Fig. 2A-G). Following acute WS exposure, all brain mRNA gene expression levels tested in this study (IL-1β, CCL-2, TNF-α, CXCL-1, CCL-5, TGF-β, and IL-6) exhibited a downregulated pattern in females compared to their respective FA controls, while males experienced upregulated levels, except for CCL-2. Only IL-1β, CXCL-1, TGF-β and IL-6 mRNA genes expressed in the brain hemispheres were notably influenced by sex and the interaction between sex and treatment (see Supplemental Table S1(a)). After WS exposure, males demonstrated increased IL-1β and TGF-β brain mRNA gene expression, while females showed a reduction, with a statistically significant difference in relative fold change (**P < 0.01, and *P < 0.05, respectively, Fig. 2A, F), according to Tukey’s post-hoc test, which implies a sex-dependent response to IL-1β and TGF-β following acute WS exposure. Moreover, a significant elevation in CXCL-1 levels was noted in male mice following acute WS exposure (*P < 0.05, Tukey’s post-hoc test), whereas no such effect was observed in female mice (Fig. 2D), which suggests distinct sex-specific phenotype and that brain mRNA gene expression responses are impacted after acute WS exposure. In response to acute WS exposure, CXCL-1 demonstrated significant differences between males and females (**P < 0.01 for males-WS versus females-WS, Tukey’s post-hoc test, Fig. 2D). Among all the analyzed genes, CXCL-1 gene expression revealed the most remarkable phenotypic differences between male and female mice in response to acute WS exposure in dissected brain hemispheres.

In the second set of studies, WS-exposed OVX mice exhibited modestly significant differences (*P* = 0.05) in hippocampal TGF-β, relative to Sham-WS exposed female mice (Fig. 3B). Notably, the expression levels of hippocampal TGF-β were also affected by surgery and the interaction between surgery and treatment (*P* = 0.05, see Supplemental Table S1(b)). The data demonstrated that neither OVX surgery, exposure treatment, nor the interaction between OVX surgery and treatment significantly altered the expression levels of the other mRNA genes examined in the hippocampus (IL-1β, TNF-α, CCL-5, CCL-2, CXCL-1, and IL-6, as shown in Supplemental Table S1(b)). After acute WS exposure in OVX mice, all hippocampal mRNA gene expression levels decreased compared to their corresponding control FA, except for CCL-5, albeit not significantly (Fig. 3A–G). This observation highlights the nuanced patterns of hippocampal mRNA gene response to acute WS exposure in OVX mice, which reveal subtle variations without statistically significant distinctions.

A noteworthy difference in inflammatory responses was observed in the cortical CCL-2 gene expression levels in OVX mice after acute WS exposure compared to their FA-controls, a trend was not evident in Sham mice (*P < 0.05, Fig. 4B). Ovariectomization led to a notable upregulation in the gene expression of both CCL-2 and CXCL-1 in the cortex following acute WS exposure, whereas Sham mice demonstrated a downregulated pattern, with a statistically significant difference in relative fold change (OVX-WS versus Sham-WS, **P < 0.01 and *P < 0.05 for cortical CCL-2 and CXCL-1, respectively, Fig. 4B, E), which signified a distinct ovarian hormone-dependent phenotype and a considerable impact on cortical mRNA gene expression responses after acute WS exposure. Particularly, in comparison to other mRNA genes analyzed in the cortex (IL-1β, TNF-α, IL-6 and CCL-5, Fig. 4A, C, D, F), only the cortical CCL-2 and CXCL-1 were significantly influenced by ovariectomy surgery and the interaction between surgery and treatment (*P* = 0.01, and *P* = 0.04, respectively, see Supplemental Table S1(c)).

### Glial fibrillary acidic protein (GFAP) staining in the cortex, dentate gyrus, and *hippocampus* following acute WS exposure

**Figure 5:**
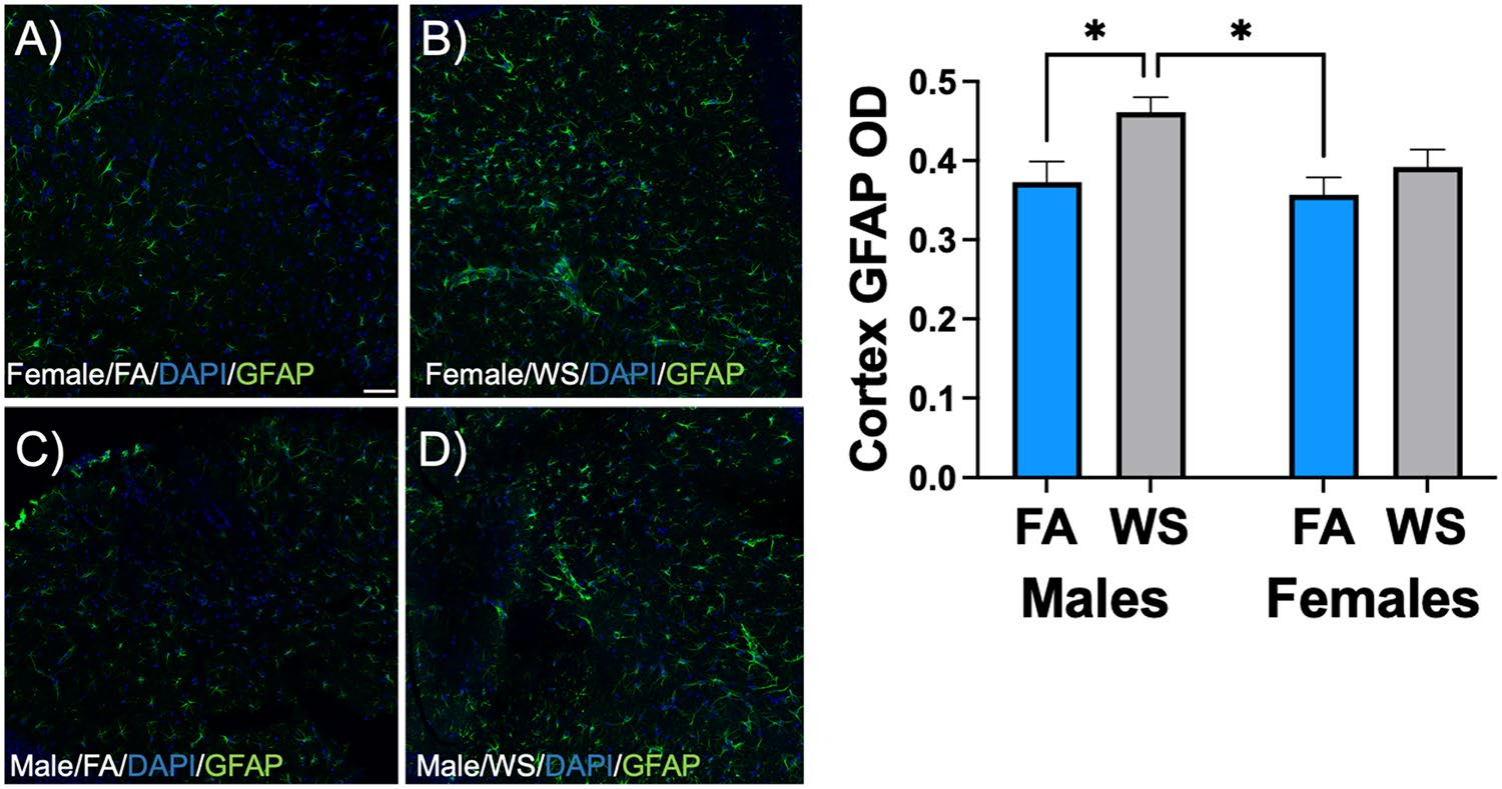
Sex-specific modulation of glial fibrillary acidic protein (GFAP) staining in the cortex of (**A**, **B**) female and (**C**, **D**) male C57BL/6 mice following acute WS exposure. The morphology of astrocytes and cell nuclei in the cortical brain regions were visualized by GFAP (green) and DAPI (blue), respectively, and were captured at excitation peaks of 490 nm and 359 nm, and emission peaks at 535 nm and 457 nm, with a scale bar of 50 µm (bottom right corner of 5A). Normalized GFAP optical density (OD) in the cortex was analyzed using a two-way ANOVA. The *P*-values for each variable (sex, treatment, and interactions between sex and treatment) concerning the cortical brain regions and GFAP marker, where *P* < 0.05 signified statistical significance, were presented in the Supplemental Table S1(d). Asterisks denoted statistically significant mean differences between groups, as identified by Tukey’s post-hoc test (*P < 0.05). Data were presented as mean normalized values ± SEM. Each treatment group consisted of *n* = 8 mice.

Further analysis of the optical density (OD) of GFAP in the cortex revealed that, after acute WS exposure, GFAP-stained astrocytes in male C57BL/6 mice exhibited a more reactive morphology indicative of activated astrocytes compared to GFAP-positive astrocytes in female mice (Fig. 5B, D). The cortical astrocytic morphology in male mice, following acute WS exposure, exhibited a higher intensity of GFAP expression compared to their respective FA-control groups (Fig. 5A–D, and Supplemental Fig. S1). Quantitative analysis of GFAP staining in brain cortex sections demonstrated that, upon acute exposure to WS, male mice exhibited a marked elevation in the cortical astrocyte OD surpassing that of the FA-exposed control group, while this alteration was not observed in female mice (*P < 0.05, Fig. 5, and see Supplemental Table S1(d)). GFAP staining in the female cortex showed a modest increase in the acute WS-exposure group, albeit not reaching statistical significance. These findings highlight sex-related variations and indicate a sex-specific impact on neurobiological responses to acute WS exposure, particularly evident in the modulation of astrocyte activation in the cortex.

**Figure 6:**
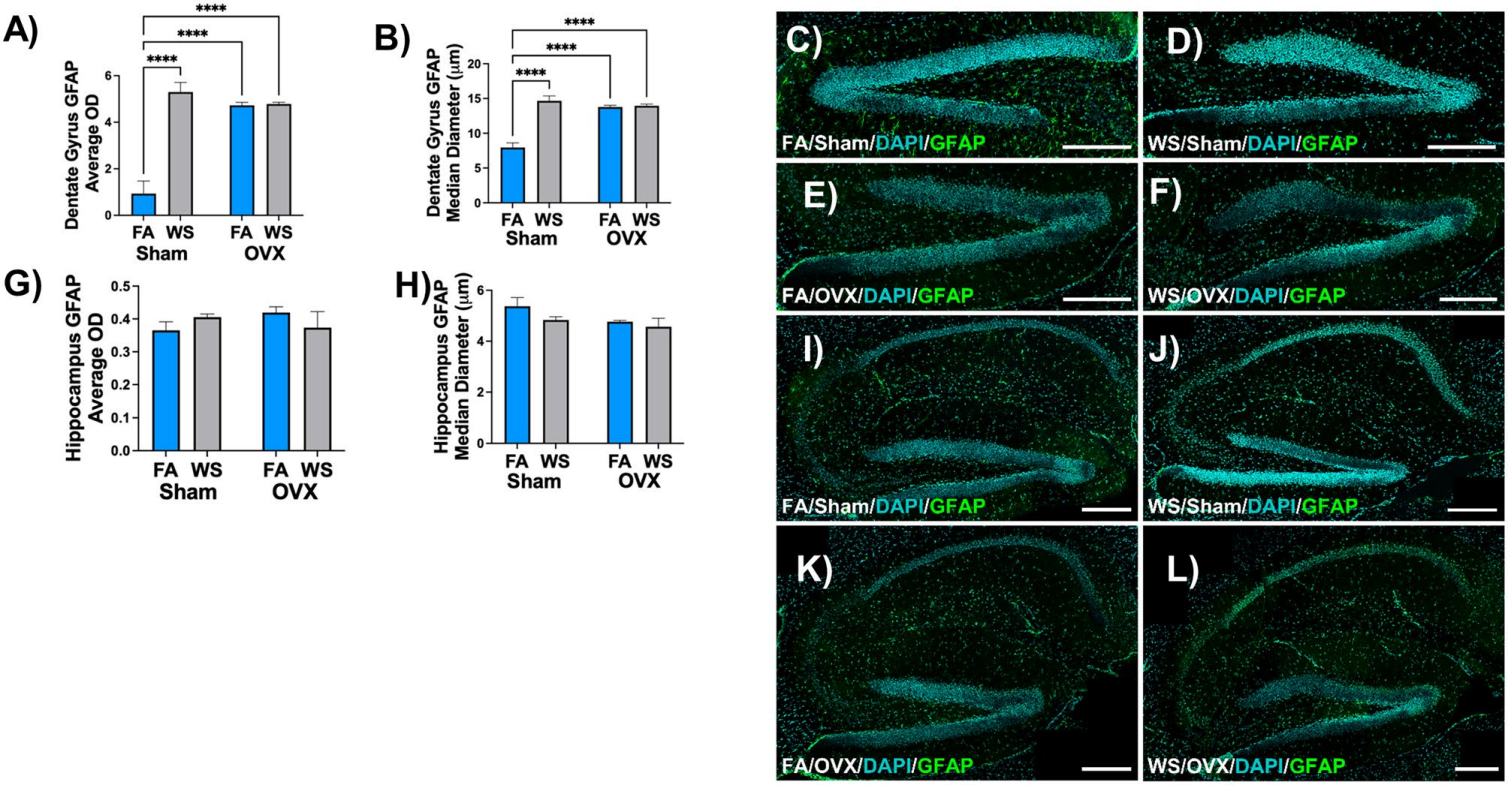
Ovarian hormone-dependent alterations in glial fibrillary acidic protein (GFAP) staining within the dentate gyrus and hippocampus of female C57BL/6 mice following acute WS exposure. (**A**, **B**) Dentate gyrus GFAP quantification with associated representative images (**C-F**), and (**G**, **H**) hippocampus GFAP quantification with corresponding representative images (**I–L**) in Sham-operated and OVX female mice exposed to control FA and acute WS. Normalized GFAP optical density (OD) in the dentate gyrus and hippocampus were analyzed using a two-way ANOVA. The *P*-values for each variable (surgery, treatment, and interactions between surgery and treatment) regarding the dentate gyrus and hippocampal brain regions and GFAP marker, where *P* < 0.05 signified statistical significance, were presented in the Supplemental Table S1(e). Asterisks denoted statistically significant mean differences between groups, as identified by Tukey’s post-hoc test (****P < 0.0001). Data were presented as mean normalized values ± SEM. The morphology of astrocytes and cell nuclei in the dentate gyrus and hippocampal brain regions were visualized by GFAP (green) and DAPI (blue), respectively, and were captured at excitation peaks of 490 nm and 359 nm, and emission peaks at 535 nm and 457 nm, with a scale bar of 250 µm. Each treatment group consisted of *n* = 8 mice.

Glial fibrillary acidic protein staining in subregions of the brain indicated significant phenotypic changes in staining between treatment groups (Fig. 6). In terms of GFAP staining OD and the median diameter of astrocytes in the dentate gyrus, a marked distinction was evident exclusively in the Sham mice group following acute WS exposure compared to their respective FA control, while under the FA control condition, OVX mice exhibited significantly higher levels than Sham mice (****P < 0.0001, Fig. 6A, B). These observations align with confocal images, which revealed a higher intensity of GFAP expression in astrocytic morphology in the dentate gyrus brain regions of Sham mice compared to OVX mice, irrespective of exposure to control FA or acute WS (Fig. 6C–F, and Supplemental Fig. S1). In contrast, there were no significant differences in the quantified hippocampal GFAP staining between Sham and OVX female C57BL/6 mice following acute WS exposure, or when compared to their respective control groups exposed to FA (Fig. 6G, H). Consistent with the GFAP quantification data (Fig. 6G, H), confocal microscopy images revealed no discernible differences in GFAP staining between Sham and OVX mice, regardless of exposure to control FA or acute WS treatment when evaluating the entire hippocampal region (Fig. 6I–L, and Supplemental Fig. S1). The results highlighted a substantial impact on the quantified parameters of average OD and astrocytic median diameter in the dentate gyrus brain regions, influenced by a combination of factors including ovariectomy surgery, exposure treatment, and the interaction between ovariectomy surgery and treatment (*P* ≤ 0.0001, see Supplemental Table S1(e)), while these parameters in the hippocampal territory remained unaffected by the same factors.

### Targeted lipidomics in the plasma and brain after acute WS exposure

**Figure 7:**
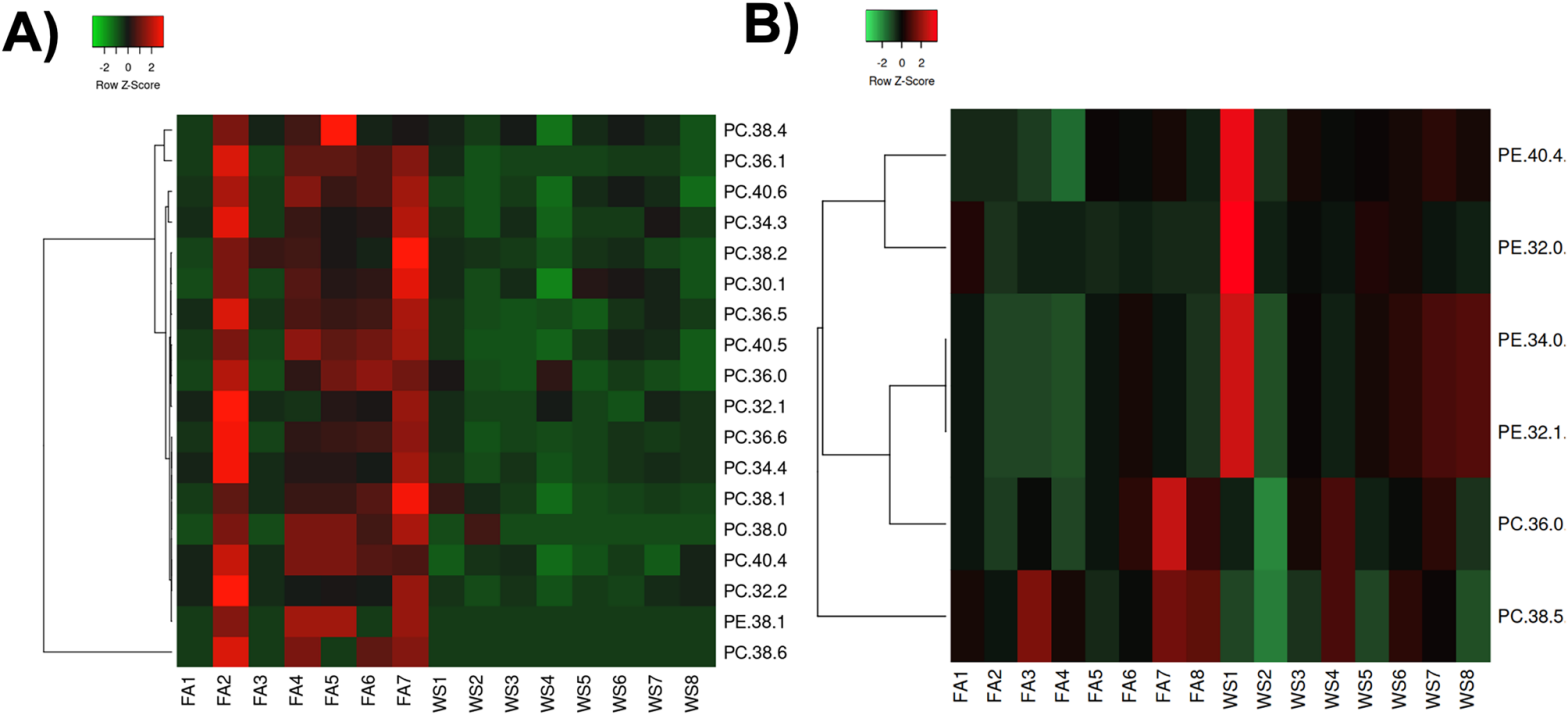
Relative expression of phosphatidylethanolamine (PE) and phosphatidylcholine (PC) lipids in plasma from female and male C57BL/6 mice. (**A**) heatmap of significant plasma lipid changes in female mice exposed to control FA (*n* = 7) and acute WS (*n* = 8), (**B**) significant plasma lipid changes in male mice. Statistics were calculated with a Mann–Whitney test and *P ≤ 0.05 was considered statistically significant.

From the lipids assessed in the plasma, female C57BL/6 mice had significant decreases following acute WS exposure in seventeen phosphatidylcholine (PC) lipids and one phosphatidylethanolamine (PE) lipid (Fig. 7A). In the plasma, male C57BL/6 mice demonstrated significant decreases in two PC lipids, PC (36:0, 38:5) (Fig. 7B). Four PE lipids (32:0, 32:1, 34:0, and 40:4) showed significant increases in the male mice, acute WS-exposed plasma, compared to the male-FA group. Overall, female mice demonstrated more phenotypic changes in plasma-lipids tested compared to male mice (18 differentially expressed PC and PE lipids, compared to only 6 PC and PE lipids in males).

**Figure 8:**
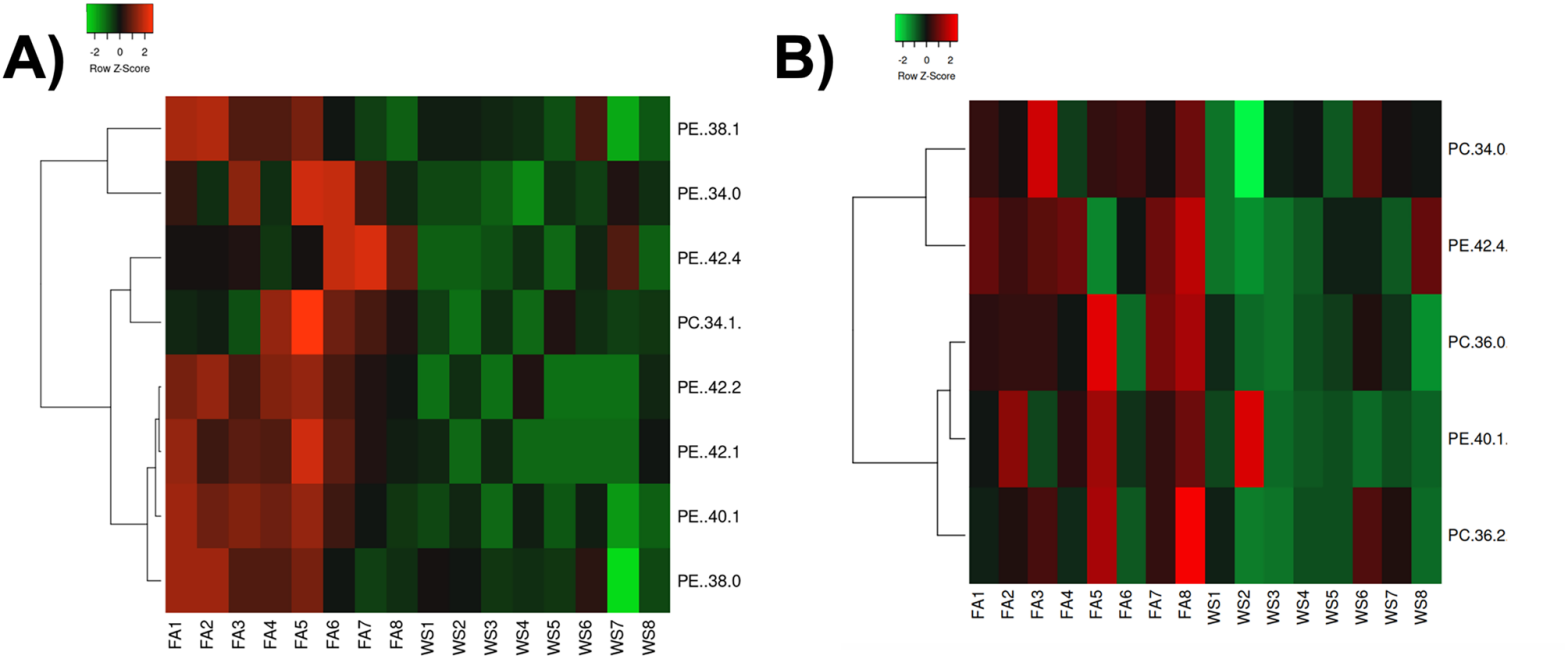
Relative expression of phosphatidylcholine (PC) and phosphatidylethanolamine (PE) in acute WS- and control FA-exposed C57BL/6 mouse brains. Compared to control FA-exposed mice, acute WS exposure significantly downregulated (**A**) PC and PE brain lipids in female mice, (**B**) PC and PE brain lipids in males. Statistics were calculated with a Mann–Whitney test and *P* ≤ 0.05 was considered statistically significant. Heatmaps display only significantly differentially expressed lipids. Each treatment group consisted of *n* = 8 mice.

Targeted lipidomics in the brain indicated a similar phenotype, with eight significantly decreased PE and PC lipids in female mouse brains, following acute WS exposure (Fig. 8A). Male mice also consistently demonstrated a decrease in PC and PE lipids following acute WS exposure including PC (34:0), PC (36:0), PC (36:2), PE (40:1), and PE (42:4) (Fig. 8B). Consistent with the plasma lipidomics, female mice consistently showed more PE and PC changes in the brain-lipids tested than male mice. The findings highlighted a distinct impact of acute WS exposure on lipid profiles in both plasma and the brain, with notable sex-specific variations, which emphasize the importance of considering sex differences in lipidomic responses at both systemic and central nervous system levels to environmental exposures.

## Discussion and conclusions

Due to the significant increase in wildfire incidences, especially in densely populated and rural regions of the western United States^31^, this study (Fig. 1) delved into the tangible consequences of this escalating phenomenon on ambient air quality. However, in recent years, exacerbated by climate change, there has been a noteworthy surge in wildfire events in North America and globally^32^. Particulate matter (PM), notably PM_2.5_^33^, which possesses a strong potential for adsorbing toxic metals and causing adverse physiological effects upon inhalation by the human body^22,34^, has actually improved in the United States since the 1980s, except in areas where wildfire emissions are prolific^33^. Building upon our previous study investigating systemic immunological responses following acute inhaled WS with metal-particulates^35^, the observed consistent increases in all detected metals within the acute WS-exposed group align directly with the broader narrative of intensified wildfire events, which emphasized their profound impact. Particularly, the metals analysis of PM revealed statistically elevated concentrations of ^63^Cu, ^182^W, ^208^Pb, and ^238^U following acute WS exposure^35^, offering in-depth insights into the specific metal components most impacted by the changing dynamics of wildfire occurrences in the region. While much of the existing air pollution literature has largely focused on urban sources of inhaled pollutants, such as diesel exhaust^36,37^, vehicular emissions^38,39^, and other industrial sources^40^, our previous research elucidated the escalating importance of wildfires as a substantial contributor to atmospheric metal pollution^35,41^. Particulate matter exposure is linked to a wide range of diseases, including cardiovascular and respiratory conditions, and lung cancer^42^.

In previous pre-clinical studies, primarily male rodents have been utilized^43–45^. This bias in air pollution research limits a comprehensive understanding of how environmental exposures impact female rodents, compared to males. Female mice, historically considered less physiological sensitive to inhaled toxins or substances^43^, were used less frequently than male mice as a model to examine the full-scope of inflammatory consequences following air pollution exposures. In order to bridge the gap on the impact of environmental exposures, particularly wildfire events, on both female and male mice, this research investigated previously unexplored facets of neuroinflammatory consequences after an acute WS exposure episode, revealing sex-dependent responses in brain mRNA gene expression that unveil acute WS impacts (Fig. 1), expanding the focus beyond lung biology^8,35^. Following acute WS exposure, distinct sex-dependent patterns emerged in brain mRNA gene expression (Fig. 2). Females consistently exhibited downregulated expression across all tested genes (IL-1β, CCL-2, TNF-α, CXCL-1, CCL-5, TGF-β, and IL-6) compared to their respective FA controls, whereas males showed upregulation in most genes tested, except for CCL2 (Fig. 2A–G). The responsiveness of specific mRNA genes in the brain (IL-1β, CXCL-1, TGF-β, and IL-6) to acute WS exposure was notably significantly influenced by sex and the interaction between sex and treatment, leading to the identification of two significant implications: (i) the identified sex-dependent responses suggest potential variations in susceptibility to acute WS-induced neuroinflammation in males and females. For instance, after acute WS exposure, males exhibited an increase in IL-1β, CXCL-1, TGF-β, and IL-6 gene expression, whereas females showed a reduction in these genes (Fig. 2A, D, F, G). These results align with earlier studies that have reported an elevation in brain mRNA gene expression, such as IL-1β production which regulates blood–brain barrier (BBB) integrity^46^, in the brains of male rodents exposed to inhalation of diesel exhaust (DE) or ambient fine airborne PM_25_ in the midbrain and cortex^47,48^. The significant upregulation of brain mRNA levels of IL-1β, TGF-β, and IL-6 in males post-acute WS exposure, as opposed to females (Fig. 2A, F, G), closely parallels prior investigations into NO_2_ exposure^43^. Elevated levels of IL-1β, IL-6, TNF-α, and TGF-β in the male cortex following NO_2_ exposure correlated with anxiety and depression-like behaviors, while females exhibited no such changes^43^. This highlights a sex-specific vulnerability to inhaled toxins in males, which rendered them more susceptible than females. (ii) The substantial elevation in CXCL-1 in males post-WS exposure emphasizes the complexity of the sex-specific gene modulation, indicating unique neuroprotective or regulatory mechanisms. These brain mRNA genes, particularly CXCL-1 and IL-1β, play critical roles in neurotoxicity within the CNS, shaping neuronal responses^49^. Brain mRNA levels of CXCL-1 and IL-1β increase in various neurodegenerative diseases and models, including Alzheimer's, Parkinson's, ischemic stroke, traumatic brain injury, and HIV-related dementia as well as in animal models exposed to environmental neurotoxicants^49,50^, such as acute WS. In Alzheimer’s disease patients, CXCL-1 levels are elevated in cerebrospinal fluid, brain tissue, and blood monocytes^51^. In pathological brain tissue, CXCL-1 inhibits Alzheimer’s disease progression by activating the CXCR2 receptor on neurons, thereby facilitating monocytes transendothelial migration into the brain for amyloid β (Aβ) plaque elimination^52^. CXCL-1 plays dual roles in multiple sclerosis progression, exhibiting neuroprotective effects while contributing to neural tissue damage through neutrophil infiltration^53^. Elevated CXCL-1 expression in the brains of multiple sclerosis patients, primarily from activated astrocytes, contributes to neural tissue damage through the infiltration of neutrophils^53^. These findings highlight the importance of investigating sex-specific factors, including sexual dimorphism, in regulating brain mRNA gene expression, offering insights into environmental exposure-susceptibility, and herald health-related measures or brain therapeutic strategies tailored to sex-specific responses.

Ovarian hormonal factors are crucial for a comprehensive understanding of how acute WS exposure affects neuroinflammation regulatory mechanisms (Fig. 1). Ovariectomy (OVX), the classic model of surgical menopause, results in diminished circulating estrogen and progesterone, however progesterone can still be produced by the adrenal cortex^54^. The investigation of OVX-impact on cerebral mRNA expression post-acute WS exposure yielded intriguing findings in regions like the hippocampus and cortex (Figs. 3, 4). Specifically, following acute WS exposure, OVX mice exhibited a notable decrease in hippocampal TGF-β compared to Sham mice (Fig. 3B), which indicates complex interactions between ovarian hormones, neuroinflammatory responses, and environmental stressors or air pollutants, exemplified by acute WS exposure. This significant reduction implies that ovarian hormone depletion, particularly estrogen, disrupts the typical regulation of TGF-β signaling pathways or signifies an impaired capacity of the hippocampus to mitigate neuroinflammatory responses and oxidative stress induced by acute WS exposure, given the pivotal role of estrogen in upholding neuroprotective and anti-inflammatory mechanisms within the hippocampus^55^. The hippocampus has long been posited as the principal locus of estrogen's cognitive effects, with explicit memory identified as the cognitive domain most vulnerable to the decline in estrogen levels during menopause^56^. The substantial decline in hippocampal TGF-β levels in OVX mice after acute WS exposure may have potential implications for hippocampal-dependent learning and memory processes^57^, as evidenced by the critical role of TGF-β signaling pathways in synaptic plasticity, neuronal survival, and cognitive function^58^. Diminished TGF-β signaling potentializes synaptic plasticity and neuroprotection deficits in OVX mice post-acute WS exposure, which elucidates the critical function of ovarian hormones, especially estrogen, in modulating neuroinflammatory responses and brain neuroprotection^55^.

The cortical gene expression patterns unveiled distinct ovarian hormone-dependent phenotypes (Fig. 4), notably marked by the upregulation of CCL-2 and CXCL-1 in OVX mice and a converse downregulated pattern in Sham mice following acute WS exposure, with a significant difference between the two groups (Fig. 4B, E). This indicates an augmented cortical neuroinflammatory response and susceptibility of cortical tissue to neuroinflammatory stimuli triggered by acute WS exposure due to estrogen depletion. CCL-2 and CXCL-1 promote the recruitment and activation of immune cells, including monocytes, microglia, and neutrophils, contributing to host defense mechanisms within the CNS^59^, where dysregulated expression of these chemokines has been linked to cognitive deficits and the pathogenesis of neurological disorders such as Alzheimer's disease, Parkinson's disease, multiple sclerosis, and other neurodegenerative diseases^59^. In Alzheimer's disease, dysregulated neuroinflammation, marked by overactivation microglia and pro-inflammatory cytokine release, contributes to neuronal damage and cognitive decline^60^. Similarly, in Parkinson's disease, neuroinflammation is associated with the loss of dopaminergic neurons^61^. In multiple sclerosis, autoimmune attacks on the CNS involve CCL-2 and CXCL-1, recruiting immune cells and causing demyelination and neurological deficits^59^. A notable disparity in neuroinflammatory responses was elucidated in the cortical CCL-2 gene expression levels of OVX mice following acute WS exposure compared to their control FA, a phenotype absents in Sham mice (Fig. 4B). The distinct expression patterns of CCL-2 and CXCL-1 in OVX mice cortex after acute WS exposure, compared to FA-control (Fig. 4B, E), suggest their roles in neuroinflammation, possibly involving inflammatory pathways or immune cell recruitment due to wildfire exposure and estrogen depletion during a menopausal state. Ovarian hormone’s influence on hippocampal and cortical mRNA expression provides insights for future studies in neurobiology and environmental health^62^. For instance, investigating hippocampal TGF-β changes could clarify BBB integrity’s role in neurotoxicity concerns during ovarian hormone deficiency or menopause, particularly regarding air pollution^63^. Further exploration of molecular mechanisms is necessary to understand the observed disparities in hippocampal and cortical responses^49,64^. These phenotypic differences emphasize the importance of considering brain region-specific effects when examining the impact of OVX surgery and environmental stimuli on brain mRNA gene expression.

The analysis of glial fibrillary acidic protein (GFAP, a marker of astroglial injury or reactivity) staining in the cortex following acute WS exposure demonstrated sex-related variations in the neurobiological responses of male and female C57BL/6 mice (Fig. 5). Astrocytes in the cortex of male mice exhibited heightened activation or morphological changes in response to acute WS exposure compared to females. Both sexes showed increased GFAP expression in cortical brain regions following acute WS exposure (Fig. 5A–D), akin to responses observed with other stressors like diesel exhaust and traffic-related air pollution^39,65^. This sex-specific difference was further accentuated by a notable increase in the optical density (OD) of GFAP in cortical astrocytes of male mice, surpassing that of the FA-exposed control group, whereas no such alteration was observed in female mice (bar chart in Fig. 5). The female cortex showed a modest increase in GFAP staining OD in the acute WS-exposure group, although it did not reach statistical significance. This study pinpointed a new dimension: sex-specific astrocyte reactions in the cortical region following wildfire exposure, further elucidating previous connections between environmental pollutant-exposure and neurobiological responses^62,66–68^. The escalated astrocytic activation in males, evidenced by a significant increase in cortical GFAP OD expression (bar chart in Fig. 5), suggested a potential predisposition to a more robust neuroinflammatory milieu in male mice compared to their female counterparts in response to acute WS exposure. Insights from astrocyte activation and neuroinflammation studies provide valuable perspectives on how environmental pollutants impact the cortical sphere^69,70^, illuminating sex-specific astrocyte responses and neuroinflammatory outcomes post-acute WS exposure. This understanding informs crucial research, laying the groundwork for tailored strategies to enhance neurological health and deciphering essential relevant molecular mechanisms^64^.

The substantial impact observed in GFAP staining within the dentate gyrus of Sham mice after acute WS exposure, in contrast to their FA controls (Fig. 6A, B), indicates a significant effect on astrocytic morphology, consistent with the established scientific understanding that environmental exposures, such as PM_2.5_ and arsenic exposure, have been shown to elicit significant alterations in astrocytic activity, affecting the dentate gyrus^71,72^. There was increased dentate gyrus GFAP OD and greater median diameter in OVX mice under FA conditions induced by OVX surgery^73^, especially when compared to the levels observed in Sham mice under similar conditions. Ovariectomy (OVX)-related hormonal changes are known to influence neural dynamics^73^, but there is less information specifically focused on the impact on astrocytic morphology in the dentate gyrus. In contrast to the distinct astrocytic alterations in the dentate gyrus, the responses of astrocytes in the total hippocampal formation showed no discernible differences in GFAP OD and GFAP median diameter levels (Fig. 6G, H) among treatment groups. These brain region differences demonstrate the necessity for a more detailed examination of astrocytic activation patterns, considering the distinctive characteristics and functions of each dentate gyrus and hippocampal formation subregion. This evidence offers a perspective on how ovarian hormones respond to acute WS exposure, enhancing our understanding of phenotypic changes in astrocytic activity. Given the critical role of astrocytes in the dentate gyrus in memory and learning processes, influencing synaptic plasticity and neuronal function^74^, the alterations discerned in astrocytic morphology bear significance, offering valuable perspectives into the potential ramifications of acute WS exposure on cognitive processes in this particular brain region. This viewpoint suggests the need to investigate immediate neuroinflammatory responses and long-term cognitive implications of altered astrocytic activity^75^. The multifaceted impact of OVX surgery, exposure treatment, and the statistical interaction on quantified GFAP parameters such as average OD and median diameter of astrocytes in the dentate gyrus, while these measures in the hippocampal territory remained unaffected by the same factors (see Supplemental Table S1(e)), suggests the need for further study into the cause of this phenomenon. Future research should focus on exploring the biochemical mechanisms driving the identified alterations and investigating the temporal aspects of astrocytic responses. This study enhances understanding of the impact of environmental stressors or air pollutants^62,68^, particularly acute exposure to WS, on astrocytic activity. Challenges for future research include examining brain region differences, hormonal and sex-specific influences, and long-term cognitive implications. Oxidative stress is pivotal in neurodegenerative diseases, impacting lipid metabolism^76–78^. The effects of external factors such as acute WS exposure on oxidative stress and lipid metabolism is currently understudied. Our study reveals phenotypic alterations in neuroinflammation between male and female mice following an acute WS exposure event. The plasma lipidome is significantly dysregulated in neurological diseases such as Alzheimer’s disease^79^. Polyunsaturated fatty acids (PUFA) PC species have been reported to be decreased in preclinical Alzheimer’s disease plasma^80^. In addition, ovarian hormone presence could exert a protective role in mRNA gene expression in the brain following acute WS exposure. Epidemiological evidence suggests that individuals are more susceptible to cardiopulmonary events following acute wildfires^81,82^, however less is known regarding neurological sequelae following wildfire events.

Woodsmoke (WS) studies and sex differences have largely focused on lung biology^8^. Long-term smoke exposure decreased the FEV (forced expiratory volume in one second)/FVC (forced vital capacity) ratio in both exposed males and females^83^. The reduction of FVC, FEV_1_ and peak expiratory flow rate (PEFR) values was present in both male and females exposed to WS. Woodsmoke PM alters epithelial immune responses in a sex-dependent manner, suppressing host defense to SARS-CoV-2 infection, using a primary human nasal epithelial cell model^8^. Data suggest that exposure to environmentally relevant PM_2.5_ depletes ovarian follicle reserve and causes sex-dependent cardiovascular changes in ApoE^−/−^ mice^84^.

Across both brain and plasma samples, female mice demonstrated robust phenotypic lipidomic changes to WS-exposure more than male mice (Figs. 7, 8). Chronic exposure to traffic-related air pollution has been shown to reduce lipid mediators including linoleic acid and soluble epoxide hydrolase in the serum of female rats^85^. Air pollution exposures have been documented to alter lipid metabolism^22^. Pro-resolving lipid mediators regulate ozone-induced pulmonary and systemic inflammation^86^. Specific compounds such as prohibitins can regulate macrophage fatty acid composition, plasma membrane packing and lipid raft-mediated inflammatory signaling^87^. Other mediators such as TREM2 have demonstrated dysregulation in the neuroinflammatory phenotype following diesel exhaust exposure^44^. Estrogen and other sex hormones could influence lipid metabolism^88,89^. Research is ongoing on how air pollution impacts endocrinological outcomes^90^. Epidemiological studies suggest that older women could be more susceptible to air pollution, yet they often overlook menopausal factors in addition to age^91,92^. One study found that estradiol mitigated ozone-induced brain lipid peroxidation and impaired social recognition memory in female rats, without addressing neuroinflammatory outcomes^93^. Overall, this study is one of the first to fully evaluate the mechanistic role of ovarian hormone presence on neuroinflammation following acute WS exposure. There are defined neurological risks that come with hormone replacement therapy during menopause, such as Parkinson’s disease^94^. Further studies exploring the mechanistic role of estrogen and progesterone following acute WS exposure will help determine windows of toxicological susceptibility throughout lifespan. However, certain limitations warrant consideration for future research. While the study primarily explores brain mRNA gene expression, an opportunity to enhance these findings arises by investigating complementary protein expression levels. Conducting an in-depth analysis of markers related to oxidative stress, such as 8-oxo-dG, and proteins associated with specific pathways like NF-κB^95^, may provide detailed insight into the functional repercussions stemming from the identified gene regulation in the brain, particularly in response to acute WS exposure. Studying epigenetic changes in affected brain areas, sex differences, and hormonal imbalances can reveal key regulatory mechanisms affecting gene expression in the brain. By investigating how genes related to neuroinflammation are affected by epigenetic marks after exposure to stress, a better understanding of the molecular dynamics influencing neuroinflammatory responses is gained^64^. For instance, elucidating these molecular mechanisms is critical for understanding the potential correlation between WS exposure and cognitive dysfunction, including Alzheimer’s disease dementia^49,64^. Incorporating single-cell RNA sequencing and spatial transcriptomics for a detailed characterization of specific cell types, following acute WS exposure, could advance knowledge of the spatial distribution of gene expression changes within brain regions. This approach would be beneficial for deciphering specific cellular responses, providing characterization of individual cell types, and elucidating their contributions to the identified neuroinflammatory patterns. Future studies may integrate additional proteomic techniques with the brain mRNA gene expression analysis conducted in this study. Specifically, we plan to utilize mass spectrometry (MS)-based quantitative proteomics to characterize the protein composition of brain tissue samples from male and female mice post-WS exposure. This enables precise identification and quantification of proteins involved in neuroinflammatory pathways, revealing sex-specific response mechanisms. In addition, longitudinal research beyond acute exposure impacts to assess the effect of chronic or repeated WS exposures would offer an in-depth exploration of the long-term outcomes and WS- cumulative effects on cognitive function and neuroinflammation^68^. A thorough examination of the hormonal aspect could involve studies which investigate methods, including pharmaceutical agents or alternative interventions, to balance or control hormones and examine the mechanistic roles of estrogen and progesterone after acute WS exposure, which is crucial for determining windows of toxicological susceptibility throughout the lifespan. In summary, these integrative methodologies not only contribute to elucidating sex-specific neuroinflammatory responses and the repercussions of ovarian hormone deficiency but also lead to avenues for further research. Future studies may also examine targeted therapeutic strategies for the brain and facilitate proactive measures against the adverse impacts of environmental pollutants like wildfires.

### Supplementary Information

Supplementary Information.

## Data Availability

The data presented in this study are available upon reasonable request from the corresponding author, Dr. Katherine Zychowski (kzychowski@salud.unm.edu).
