# Supplementary material for: Neuroinflammation is dependent on sex and ovarian hormone presence following acute woodsmoke exposure
Source: Sci Rep. 2024 Jun 6;14:12995. doi: 10.1038/s41598-024-63562-2 (PMC11156661; doi:10.1038/s41598-024-63562-2)
Supplement: Supplementary file 1 — Supplementary Information. [file 41598_2024_63562_MOESM1_ESM.docx]

**Supplemental** **Figure S1.** **HALO example assignment and automated analysis.** All images were uploaded onto HALO analysis software. DAPI (blue) stained objects were classified as object 1. GFAP (green) stained objects were classified as object 2. Objects were first distinguished based off color. Objects were then gated based on size. Objects too large or small to fall within the size range of our targeted objects were not analyzed in the final images. Once analyzed object 1 became red and object 2 remained green in the post analysis image.

**Supplemental Figure S2. Verification and validation of lipidomic screening.** Total phosphatidylcholine (PC) verification in female mouse plasma (n=6-10 per treatment group). Statistics were determined with a Student’s T-test and * indicate *P ≤ 0.05*. Results are consistent with high-throughput lipidomics screening outlined in Figure 7.


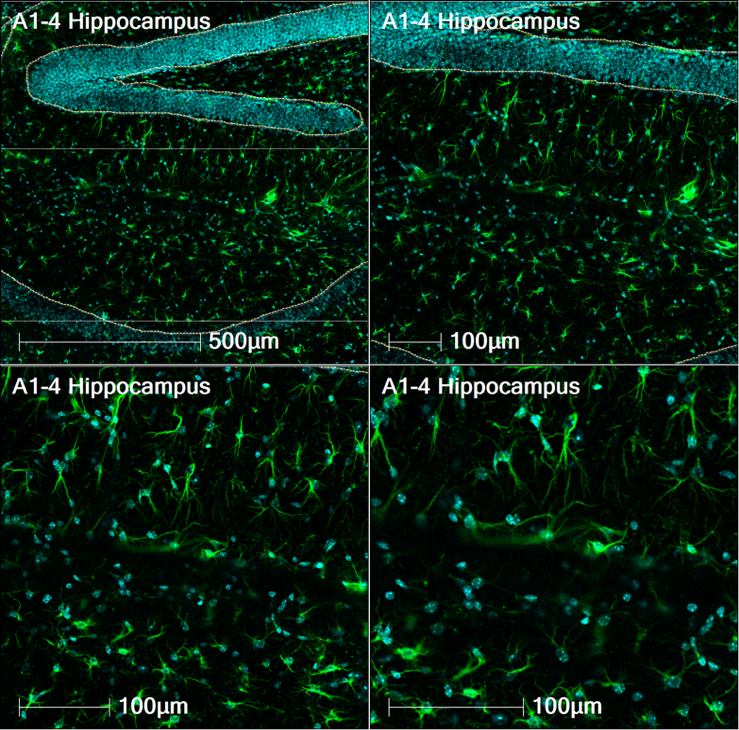

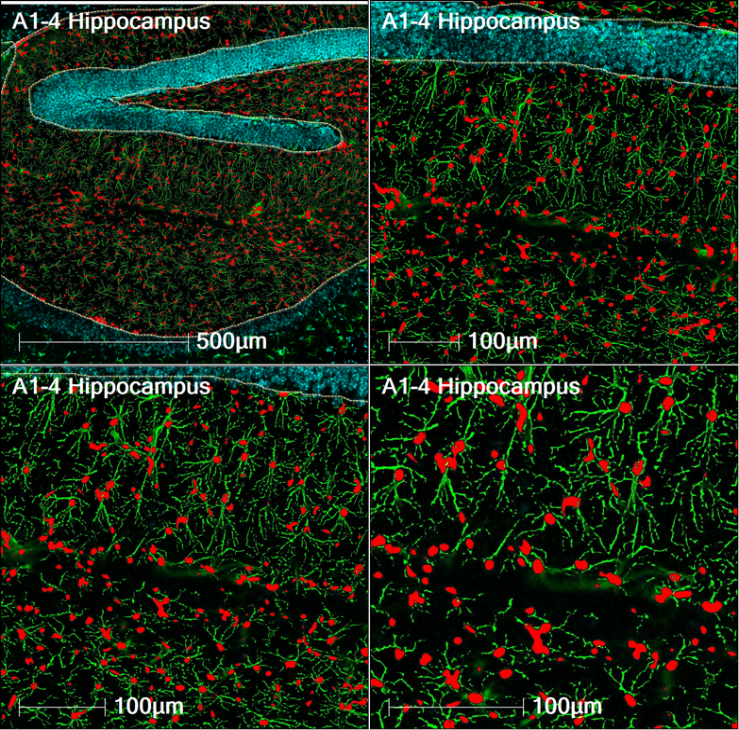


**Supplemental** **Figure S1. HALO example assignment and automated analysis.** All images were uploaded onto HALO analysis software. DAPI (blue) stained objects were classified as object 1. GFAP (green) stained objects were classified as object 2. Objects were distinguished based on assigned color. Objects were then gated based on size. Objects too large or small to fall within the size range of our targeted objects were not analyzed in the final images. Once analyzed object 1 became red and object 2 remained green in the post analysis image.

**Supplemental Table S1*. P-values* for statistical analysis of acute WS exposure effects on various variables:** (**a**) Analysis of sex-dependent brain mRNA gene expression levels in response to acute WS exposure accounted for sex, treatment, and their interactions. (**b**) The effects of OVX on hippocampal mRNA gene expression levels involved attention to surgery, treatment, and interactions between surgery and treatment. (**c**) Ovarian hormone-dependent responses of mRNA gene expression in the cortex considered surgery, treatment, and interactions between surgery and treatment. (**d**) Sex-specific modulation of cortical brain regions and GFAP marker expression considered sex, treatment, and interactions between sex and treatment. (**e**) Ovarian hormone-dependent alterations in dentate gyrus and hippocampal brain regions and GFAP marker expression involved consideration of surgery, treatment, and interactions between surgery and treatment. Statistical significance was determined at **P < 0.05*. Each treatment group consisted of *n=8* mice.

**Supplemental Figure S2. Verification and validation of lipidomic screening.** Total phosphatidylcholine (PC) verification in female mouse plasma (n=6-10 per treatment group). Statistics were determined with a Student’s T-test and * indicate *P ≤ 0.05*. Results are consistent with high-throughput lipidomics screening outlined in Figure 7.

**Supplemental Table S1(a).** Analysis of sex-dependent brain mRNA gene expression levels in response to acute WS exposure accounted for sex, treatment, and their interactions. Statistical significance was determined at *P < 0.05. Each treatment group consisted of n=8 mice.

| **Cytokine** | **Sex**  **(M, F)** | **Treatment**  **(FA, WS)** | **Interaction**  **(Sex x Treatment)** |
| --- | --- | --- | --- |
| IL-1β | **0.01*** | 0.73 | **0.01*** |
| CCL-2 | 0.56 | 0.19 | 0.56 |
| TNF-α | 0.06 | 0.41 | 0.06 |
| CXCL-1 | **<0.01*** | 0.28 | **<0.01*** |
| CCL-5 | 0.16 | 0.83 | 0.16 |
| TGF-β | **0.02*** | 0.78 | **0.02*** |
| IL-6 | **0.01*** | 0.79 | **0.01*** |

**Supplemental** **Table S1(b).** The effects of OVX on hippocampal mRNA gene expression levels involved attention to surgery, treatment, and interactions between surgery and treatment. Statistical significance was determined at *P < 0.05. Each treatment group consisted of n=8 mice.

| **Cytokine** | **Surgery**  **(Sham, OVX)** | **Treatment**  **(FA, WS)** | **Interaction**  **(Surgery x Treatment)** |
| --- | --- | --- | --- |
| IL-1β | 0.65 | 0.13 | 0.65 |
| TGF-β | **0.05*** | 0.17 | **0.05*** |
| TNF-α | 0.07 | 0.99 | 0.07 |
| CCL-5 | 0.96 | 0.73 | 0.96 |
| CCL-2 | 0.30 | 0.36 | 0.30 |
| CXCL-1 | 0.96 | 0.30 | 0.96 |
| IL-6 | 0.89 | 0.63 | 0.89 |

**Supplemental Table S1(c).** Ovarian hormone-dependent responses of mRNA gene expression in the cortex considered surgery, treatment, and interactions between surgery and treatment. Statistical significance was determined at *P < 0.05. Each treatment group consisted of n=8 mice.

| **Cytokine** | **Surgery**  **(Sham, OVX)** | **Treatment**  **(FA, WS)** | **Interaction**  **(Surgery x Treatment)** |
| --- | --- | --- | --- |
| IL-1β | 0.20 | 0.22 | 0.20 |
| CCL-2 | **0.01*** | 0.16 | **0.01*** |
| TNF-α | 0.13 | 0.89 | 0.13 |
| IL-6 | 0.07 | 0.72 | 0.07 |
| CXCL-1 | **0.04*** | 0.37 | **0.04*** |
| CCL-5 | 0.17 | 0.99 | 0.17 |

**Supplemental Table S1(d).** Sex-specific modulation of cortical brain regions and GFAP marker expression considered sex, treatment, and interactions between sex and treatment. Statistical significance was determined at *P < 0.05. Each treatment group consisted of n=8 mice.

| **Brain region and GFAP marker** | **Sex**  **(M, F)** | **Treatment**  **(FA, WS)** | **Interaction**  **(Sex x Treatment)** |
| --- | --- | --- | --- |
| Cortex Average OD | 0.07 | **0.01*** | 0.25 |

**Supplemental Table S1(e).** Ovarian hormone-dependent alterations in dentate gyrus and hippocampal brain regions and GFAP marker expression involved consideration of surgery, treatment, and interactions between surgery and treatment. Statistical significance was determined at *P < 0.05. Each treatment group consisted of n=8 mice.

| **Brain region and GFAP marker** | **Surgery**  **(Sham, OVX)** | **Treatment**  **(FA, WS)** | **Interaction**  **(Surgery x Treatment)** |
| --- | --- | --- | --- |
| Dentate Gyrus Average OD | **<0.0001*** | **<0.0001*** | **<0.0001*** |
| Dentate Gyrus Median Diameter (αm) | **<0.0001*** | **<0.0001*** | **<0.0001*** |
| Hippocampus Average OD | 0.74 | 0.93 | 0.21 |
| Hippocampus Median Diameter (αm) | 0.14 | 0.21 | 0.55 |
